# Presence and simulator sickness predict the usability of a virtual reality attention task

**DOI:** 10.1007/s10055-023-00782-3

**Published:** 2023-03-24

**Authors:** Alexandra Voinescu, Karin Petrini, Danaë Stanton Fraser

**Affiliations:** 1 Department of Psychology, University of Bath, Claverton Down, Bath, BA2 7AY UK; 2 Centre for the Analysis of Motion, Entertainment Research and Applications, University of Bath, Bath, UK; 3 International Institute for the Advanced Studies of Psychotherapy and Applied Mental Health, Babeș-Bolyai University, Cluj-Napoca, Romania

**Keywords:** Virtual reality, Usability, Performance, Attention, Presence, Simulator sickness, Mental workload

## Abstract

Attention is the ability to actively process specific information within one’s environment over longer periods of time while disregarding other details. Attention is an important process that contributes to overall cognitive performance from performing every day basic tasks to complex work activities. The use of virtual reality (VR) allows study of the attention processes in realistic environments using ecological tasks. To date, research has focused on the efficacy of VR attention tasks in detecting attention impairment, while the impact of the combination of variables such as mental workload, presence and simulator sickness on both self-reported usability and objective attention task performance in immersive VR has not been examined. The current study tested 87 participants on an attention task in a virtual aquarium using a cross-sectional design. The VR task followed the continuous performance test paradigm where participants had to respond to correct targets and ignore non-targets over 18 min. Performance was measured using three outcomes: omission (failing to respond to correct targets), commission errors (incorrect responses to targets) and reaction time to correct targets. Measures of self-reported usability, mental workload, presence and simulator sickness were collected. The results showed that only presence and simulator sickness had a significant impact on usability. For performance outcomes, simulator sickness was significantly and weakly associated with omission errors, but not with reaction time and commission errors. Mental workload and presence did not significantly predict performance. Our results suggest that usability is more likely to be negatively impacted by simulator sickness and lack of presence than performance and that usability and attention performance are linked. They highlight the importance of considering factors such as presence and simulator sickness in attention tasks as these variables can impact usability.

**Supplementary Information:**

The online version contains supplementary material available at 10.1007/s10055-023-00782-3.

## Introduction

Over 50 years since Ivan Sutherland’s first experiments with a “head-mounted three-dimensional display” (Sutherland 1965), developments in the price, accessibility and quality (e.g., better resolution, graphics and less latency) of hardware and software now make VR viable for mass-market consumption. The availability and new capabilities of VR hardware is enabling broader applications including workplace applications, film and documentaries and healthcare and well-being applications. For example, Facebook is using VR for augmented workspace in light of changes in work patterns caused by the COVID-19 pandemic (The Independent 2020). Independent film makers are using VR to create immersive documentaries such as the Waiting Room shown at major film festivals (East City Films 2021), and VR nature scenes such as the Forrest of Serenity are being developed for palliative care (Nwosu et al. 2021). An increasing number of VR applications for physical or mental well-being applications are available for use at home. Examples are Beat Saber for fitness (Beat Games 2018) or Tripp (Tripp 2020) for meditation to name only a few.

VR enables the development of highly ecological environments and interactive 3D simulations of real-world scenarios using a combination of technologies (e.g., head-mounted displays (HMDs), large screen displays, desktop computers, video capture systems, tracking systems, headphones, motion-sensing gloves, joysticks, keyboards) (Parsons et al. 2017). One important characteristic of VR is the ability to generate realistic or imaginary environments and to deliver them in a safe and controlled space (Grabowski and Jankowski 2015; Rose et al. 2005). This is one of the reasons why VR is currently being used for training programs where real-life training would be dangerous or very difficult to simulate such as: firefighting (Yu et al. 2016), driving of autonomous vehicles (Sportillo et al. 2018), surgery and medical procedures (Aim et al. 2016; Alaker et al. 2016). New VR-based systems designed for mental health practices are starting to be used with patients in hospitals and private practices. For example, VR has potential in treating phobias and to manage anxiety and pain (Freeman et al. 2018; Freeman et al. 2017; Georgescu et al. 2020; Digital Health 2020). A key factor of VR experiences is presence, also known as the feeling of actually “being there” in the virtual environment (VE) (Slater and Wilbur 1997; Witmer and Singer 1998), and is thought to enhance the user experience (Sun et al. 2015). Users who report increased levels of presence in VR pay less attention to the physical world and perceive the VR experience as a real one in which they are immersed (Slater and Wilbur 1997). A high sense of presence in VR indicates that the participants behave and perform in VR as they would normally do in a real environment (Slater and Wilbur 1997), thus enhancing ecological validity. For example, presence is essential for adequate fear responses in VR (Brade et al. 2017; Gromer et al. 2019; Price and Anderson, 2007; Rizzo et al. 2019). Increased presence is also considered to facilitate the transfer of skills acquired in VR to the real world (Slater et al. 1996) and to correlate with increased VR system usability (Brade et al. 2017; Sun et al. 2015; Wienrich et al. 2018).

The potential for cognitive engagement promised by presence has led to huge interest in VR, not just within games but also within non-fiction content. There is a diverse body of research into the experience of VR that has been conducted within psychology and human computer interaction (HCI) since the 1990s, employing a variety of methods. For example, work conducted as early as 2001 reviewed the role of presence and emotional responses in VR as the potential of VR in psychotherapy started to be explored (Rizzo et al. 1999; Schuemie et al. 2001). After more than two decades, the concept of presence in VR and its mechanisms are still being tested and reviewed in the literature in light of new developments in hardware and software (Slater et al. 2022).

### Overview of cognitive attention tasks in virtual reality

Attention is considered one of the most important processes in human cognition, and impaired attention affects overall cognitive performance even if other cognitive processes are intact (Lezak et al. 2012). In medical settings, impaired attention is one of the most common problems in mental health conditions (Lezak et al. 2012). Sustained attention or vigilance is a cognitive process that requires the ability to maintain concentration and be alert to stimuli over prolonged periods of time. Attention is important to HCI activities as well (Hitchcock et al. 1999). For example, failing to detect, respond and act on time to targets or stimuli might have severe consequences in work environments that require sustained attention (e.g., training high risk activities) (Arrabito et al. 2015).

The measurement of cognitive performance including attention is one area in which VR is being used with success mainly due to increased ecological validity because of the ability to create experiences that are very similar to those in real life and assess how people perform in highly realistic situations (Rizzo et al. 2019; Voinescu et al. 2021). Systematic reviews and meta-analyses have shown that VR is effective in assessing cognitive processes such as attention, memory, executive functioning and visuospatial analysis and detecting clinical impairment (Negut et al. 2016).

Tests designed to measure attention using VR have focused almost exclusively on assessing attention deficits in children with Attention deficit hyperactivity disorder (ADHD) or other conditions known to have impaired attention such as traumatic brain injury or neurofibromatosis type 1 (see Negut et al. 2016; Parsons et al. 2019 for reviews). Attention was measured solely with the Virtual Classroom paradigm (Rizzo et al. 2000; Iriarte et al. 2016). Within the VR classroom, attention performance is measured using the continuous performance test paradigm (CPT) embedded in the VE (see Gilboa et al. 2018; Parsons et al. 2019; Parsons and Rizzo 2019 for a review). The CPT is known as one of the most widely used and reliable measures of sustained vigilance and attention (Gualtieri and Johnson 2005; Huang-Pollock et al. 2012). The CPT embedded in the VR classroom consists of inhibition CPTs (non-X tasks) as they ask the participant to respond to the non-target stimuli and to ignore target stimuli (Negut et al. 2017). In the VR classroom, the child sits at their desk in the classroom and has a 360° view of the classroom, including peers and teacher near the blackboard. The attention task itself is a classic CPT and consists of letters that appear on the blackboard and the child has to respond to them following a given rule (e.g., press the left mouse button as quickly as possible whenever you see letter “k” but only if letter “k” comes after letter “a” and ignore all other sequence of letters) (Negut et al. 2017; Parsons and Rizzo 2019). The results from several meta-analyses are consistent and show that VR classroom tests are effective in detecting attention impairments among children with ADHD (Gilboa et al. 2018; Negut et al. 2016; Parsons et al. 2019).

One meta-analysis went further and reported not only data on clinical efficacy but synthesized and presented descriptively data on safety, enjoyment and sense of presence reported in the included studies (Gilboa et al. 2018). In terms of safety, many studies conducted to date which have used the VR classroom to assess attention among children reported no or minimal adverse effects (Adams et al. 2009; Bioulac et al. 2012; Mühlberger et al. 2020; Nolin et al. 2009, 2016; Pollak et al. 2010, 2009; Rizzo et al. 2000). A few did not report data on safety (Areces et al. 2018; Diaz-Orueta et al. 2014; Iriarte et al. 2016; Rodriguez et al. 2018), and one reported that two children experienced simulator sickness symptoms, as they reported at least one severe symptom (Negut et al. 2017). Of the above studies, one study included measures of presence and reported no significant correlations between attention performance and presence (Nolin et al. 2016). Pollak et al. (2009, 2010) included several items to measure enjoyment, success, discomfort and perceived difficulty between VR classroom and computerized CPT. The results showed that the VR classroom was rated as more enjoyable, but no significant differences emerged on success, discomfort and perceived difficulty. Negut et al. (2017) used cognitive absorption as a measure of deep involvement with software that predicts usage behavior and reported no significant differences between VR classroom and computerized CPT. Measures of usability and presence have been scarcely reported, and their potential effects on attention performance in VR are unknown.

Recently, a new measure of attention assessment in VR for adults was developed (Climent et al. 2021). Nesplora Aquarium is the first VR-based test aimed at assessing attention in adults over 18 years and follows the same CPT paradigm as the VR classroom. Participants are immersed in a VR aquarium and sit in front of a large tank. Stimuli are various species of fish that appear one at a time, and the participant task is to respond as quickly as possible by pressing a button following a specific rule (e.g., press the button when they see or hear other fish than the clown fish). It consists of inhibition CPTs (non-*X* tasks) as they ask the participant to respond to the non-target stimuli (all other fish such as surgeon) and to ignore target stimuli (e.g., clown). Studies have shown its validity and sensitivity in detecting attention deficits among populations known to have impaired attention such as people with depression and anxiety (Voinescu et al. 2021). It also can predict past and current symptoms of ADHD among adults (Areces et al. 2019). In recent work (Voinescu et al. 2021), our clinical efficacy study reported measures of presence, simulator sickness and usability. Comparison between healthy participants and people with depression and anxiety revealed that the latter rated the Nesplora Aquarium worse in terms of usability, but no differences emerged on simulator sickness and presence between groups; however, the relationship between self-report task difficulty or mental workload, presence, system usability and performance was not evaluated in this study. Another study conducted by Li et al. (2020) designed an attention task based on the Poser task where participants had to guess the location where ocean animals would appear on the screen and to press color-matched buttons if the stimuli was a target and not to release the thumb if it was a non-target. The task was adaptive and real-time feedback was offered to the participant. The study compared attention performance between tasks in VR and on the desktop among a sample of healthy participants. The results revealed that attention performance was better in VR and presence was higher in the VR condition. To summarize, variables such as usability, difficulty and presence remain still under-reported in studies that tested attention tasks in VR in healthy and clinical populations and the effects of these variables on performance on attention tasks have not been previously investigated.

### Usability and VR attention tasks

As the core component of a VR system is an advanced human computer interface  (Rizzo et al. 2019), it is essential to investigate how system usability and human factors interact with each other and may affect the VR experience and performance (de França et al. 2018). Usability refers to the characteristics of a system or product that allows the user to perform the tasks safely, effectively and efficiently while enjoying the experience (ISO 9241-11: 2018). It concerns both the system being used and the person who is interacting with that system with two broad categories of usability measures being available: objective and subjective. Objective usability (from hereinafter called performance) concerns how users perform with a specific product and subjective usability (from hereinafter called usability) measures how much users like the system (Nielsen and Levy 1994). Performance is usually assessed by quantifying the total time taken to complete a task or the number of errors made by users on a given task. Usability is measured using scales (e.g., System Usability Scale, Brooke 1986) which ask the user to evaluate the system after interacting with it (Nielsen and Levy 1994). Research has revealed positive correlations between performance and usability which suggests that users prefer systems where they perform well or perform well because of increased usability (Hornbæk and Law 2007; Nielsen and Levy 1994).

Traditionally, studies have focused on the usability of, and performance on websites, and graphical user interfaces or database management, for cognitive attention tasks in VR, our overview of studies revealed that most were designed as efficacy studies with the aim of establishing the tasks’ validity and efficacy in detecting attention impairments. For usability, most studies focused on safety by reporting simulator sickness experienced during attention tasks and only a few collected data on several variables deemed important to VR research such as self-report task difficulty or mental workload, presence, system usability (see Gilboa et al. 2018; Voinescu et al. 2021). None of the studies on attention tasks in VR assessed the impact of these variables on performance and usability.

## Related work

### Mental workload and usability of VR applications

Task difficulty has also previously used to characterise VR tasks (Neguţ et al. 2016). Subjectively, it can be measured as mental workload and assesses the way in which users interact with technology (Longo 2018). Cognitive/mental load is related to mental processing resources including attention and can be inflated by tasks (or task features) that are complex, busy environments and stressful situations (Wickens et al. 2015). Thus, either increased or reduced workload could impact human performance. Few studies have assessed the relationship between mental workload and usability in the case of immersive VR tasks. Using an immersive VR serious game for workplaces in intralogistics, Kretschmer and Terharen (2019) showed that the usability of the VR game was negatively associated with mental workload, meaning that with the increase in usability of the design mental workload decreased. Another study assessed mental workload differences among immersive VR systems such as an HMD, a desktop screen and a video projector, and the HMD and video resulted in the high mental workload reported. However, on the self-report measure of usefulness of the wheelchair training activity performed in VR participants rated VR the highest (Rivera-Flor et al. 2019).

### Presence and usability of VR applications

Presence is positively correlated with VR system usability (Brade et al. 2017; Sun et al. 2015; Wienrich et al. 2018), and this could mean that usable systems may facilitate presence. Brade et al. (2017) evaluated the usability of a geocaching game using a smartphone in a CAVE VR environment and identified a positive correlation between usability and presence in VR. Wienrich et al. (2018) also identified a positive correlation between presence and usability in a VE delivered via an HMD where participants had to perform a group repair job of a wind turbine. Similar positive relationships were reported also by Sun et al. (2015) using a Kinect-based exergame for boxing training. However, not all studies reported significant correlations between presence and usability. For example, Mondellini et al. (2018) reported no significant association between presence and usability in an immersive VR supermarket delivered via an HMD.

The relationship between presence and performance on a VR task is, however, not straightforward as findings are often mixed. Some studies identified a positive relationship between presence and performance across various environments and tasks (Cooper et al. 2018; Price et al. 2011; Stevens and Kincaid 2015). In a virtual army simulation, presence reported by soldiers was positively associated with performance (Stevens and Kincaid 2015). Participants asked to perform a wheel change simulation task in VR performed better (task completion time) when they reported higher levels of presence (Cooper et al. 2018). However, other experimental studies failed to identify a positive association between presence and performance and others identified a negative association where increased presence resulted in poorer performance (Makransky et al. 2019; Mondellini et al. 2018; Rose and Chen 2018; Slater et al. 1996).

### Simulator sickness and usability of VR applications

Studies that used VR across various platforms, participants or tasks have reported increased concern related to simulator sickness (Hutton et al. 2018; Kolasinski 1995; Matheis et al. 2007; Mousavi et al. 2013). Symptoms of simulator sickness are similar to those of motion sickness and occur during exposure in VR (e.g., general discomfort, fatigue, headache, eye strain, nausea, dizziness) (Kennedy et al. 1993; Kolasinski 1995; Stanney et al. 1997). A potential explanation for simulator sickness comes from the sensory-conflict theory and suggests that symptoms are caused by a mismatch between signals from sensory channels: visual, vestibular and proprioception (Cobb et al. 1999). Also, symptoms can be explained by both the VR setup and/or VE design and individual vulnerability (Reinhard et al. 2019). To examine the relationship between simulator sickness and usability, a number of studies used a VR driving simulator. These studies found a negative correlation between usability and simulator sickness (Schultheis et al. 2007; Sharples et al. 2007; Voinescu et al. 2020). For other VR-based platforms, the results are mixed. Using a highly immersive CAVE environment, Brade et al. (2017) reported a negative correlation between simulator sickness and VR usability. In contrast, a lack of a relationship between simulator sickness and usability ratings was reported by Wienrich et al. (2018) for a VR-based story in which the user had to repair a wind turbine using a multi-user, multi-room VR installation platform. Similarly, Mondellini et al. (2018) reported no significant association between simulator sickness and usability in an immersive VR supermarket administered using an HMD.

The scientific literature on the link between simulator sickness and performance on VR tasks is also mixed. Studies using VR driving simulators found no significant relationship between simulator sickness and driving performance (Helland et al. 2016; Mullen et al. 2010; Muttray et al. 2013). According to a recent study, the relationship between simulator sickness and task performance is more complex showing different types of associations between simulator sickness and reaction time depending on the sensory information (auditory or visual) used in the HMD VR application (Voinescu et al. 2020). Other studies that assessed reaction time immediately after VR exposure on a virtual roller coaster or a virtual bike identified negative correlations with simulator sickness (Mittelstaedt et al. 2019; Nalivaiko et al. 2015; Nesbitt et al. 2017). For working memory or mental rotation, the results are also mixed, with some studies showing a negative correlation with simulator sickness (Bos 2015; Levine and Stern 2002) and others showed no association (Mittelstaedt et al. 2019).

### Demographics and usability

A wide range of human factors have been proposed to impact the efficacy of human performance (e.g., task and user characteristics, sensory and physiological capabilities, previous experience such as playing videogames), and health and safety issues (Maneuvrier et al. 2020; Stanney et al. 1998). Individual differences, such as age, gender and experience, are important moderators in the well-known Unified Theory of Acceptance and Use of Technology (UTAUT) that predicts usage behavioral and intention with the use of novel technology (Venkatesh and Bala 2008). Age is well known to impact usability and performance (Fisk et al. 2009), and effects of age have been identified for VR applications (Fang and Huang 2021). Similarly, recent research has identified that gender could also impact VR usability and performance, as there are data to suggest that women experience VR differently to men e.g., simulator sickness, poorer fit of the VR headsets, a higher sense of discomfort in emotional/arousing simulations (see Grassini and Laumann 2020; Stanney et al. 2020).

### Current study

The study objectives are part of a larger study that was previously published by Voinescu et al. (2021) where the main aim was to assess the efficacy of Nesplora Aquarium in detecting cognitive impairment. The present study is focused on usability and its main aim to investigate if the same set of human factors are predictors of both usability and performance. Our primary hypotheses were: (1) mental workload, presence and simulator sickness would predict usability; (2) mental workload, presence and simulator sickness would predict performance in VR; and (3) performance in VR would be significantly associated with self-report usability.

## Methods

### Participants

Ninety-one participants took part in the study. Four were excluded due to data loss which was caused by a weak wireless signal during VR testing. The age of the remaining 87 participants included in the analysis ranged between 19 and 61 years (*M* = 31.81, SD = 9.78). The sample consisted of slightly more women than men (*N* = 53, 61%). Forty percent of participants reported previous VR experience (*N* = 35). Most of them had an undergraduate (*N* = 27, 31%) or post-graduate degree (*N* = 33, 38%) (see Appendix A. Supplementary materials Table 1 for participants’ demographic characteristics). Exclusion criteria included (a) a clinical diagnosis of neurological conditions, (b) a moderate to major visual or hearing impairment, (c) age under 18 years and above 65 years^1^ and (d) severe motion sickness. We were interested in examining how simulator sickness would affect usability and performance for participants who could complete the VR task, and thus, we excluded people with severe motion sickness. This is because severe motion sickness could predict severe simulator sickness (Golding et al. 2021; Kennedy and Fowlkes 1992) and consequently not only impede participants from completing the VR task but also raise ethical considerations. The mean of simulator sickness reported by our participants was 5.78 (SD = 5.13), which means that our participants reported, on average, minimal symptoms (Stanney et al. 1997).

### Virtual reality task

**Fig. 1:**
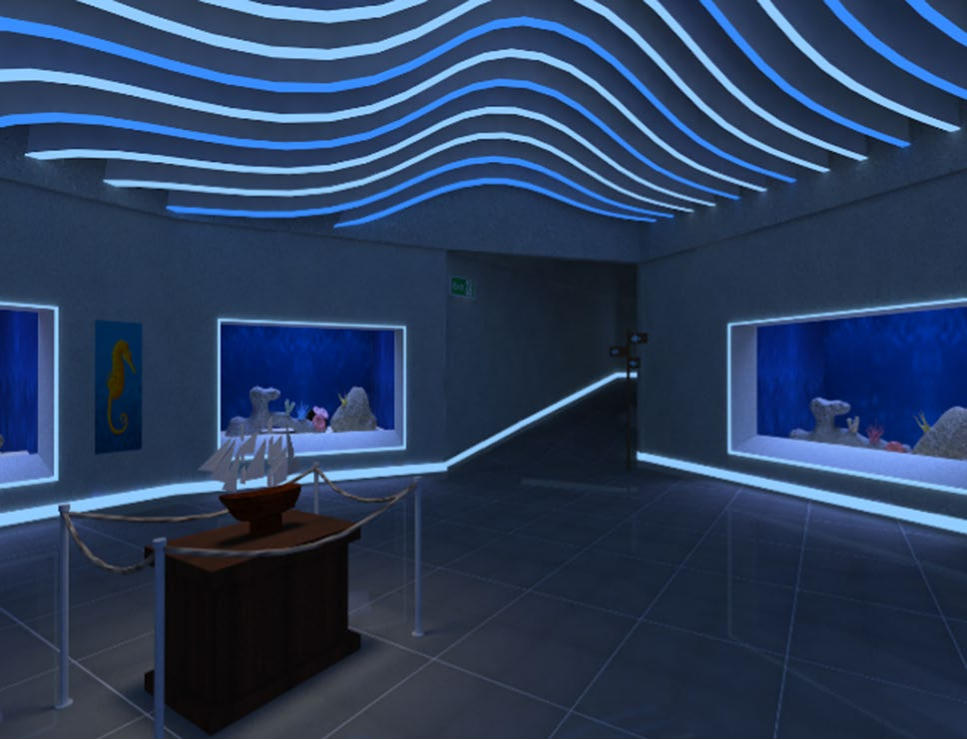
Screenshot of the virtual aquarium

**Fig. 2:**
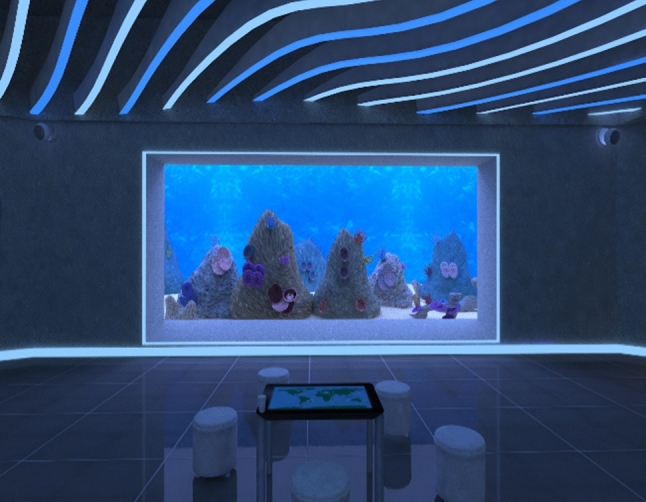
Main fish tank

Performance in VR was measured using Nesplora Aquarium a validated measure of attention (Climent et al. 2021). In Nesplora Aquarium, the virtual environment (VE) is a virtual aquarium (see Fig. 1). During the test, the participant is virtually positioned in front of the main tank of a virtual aquarium (see Fig. 2).

During the task, the participant must pay attention and respond correctly, and as fast as possible, to the stimuli following a predefined rule. The stimuli are either visual or auditory. Visual stimuli are various species of fish (e.g., clown fish, surgeon) which appear at a fast pace (500 ms) in the main tank of the aquarium. Auditory stimuli are delivered at a similar speed (approximately 700 ms) to the headphones and are the names of the fish (e.g., clown fish, surgeon). In their response, participants must follow a predefined rule, for example, each time they see any other fish than the “clown” or they hear any other fish except the “surgeon” they should press the button.

**Fig. 3:**
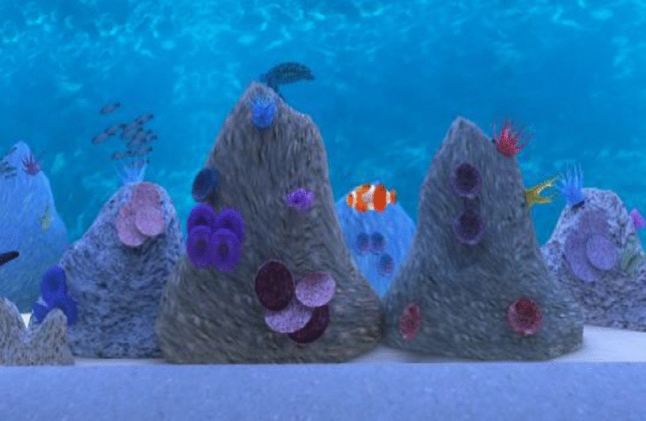
Clown fish stimuli

**Fig. 4:**
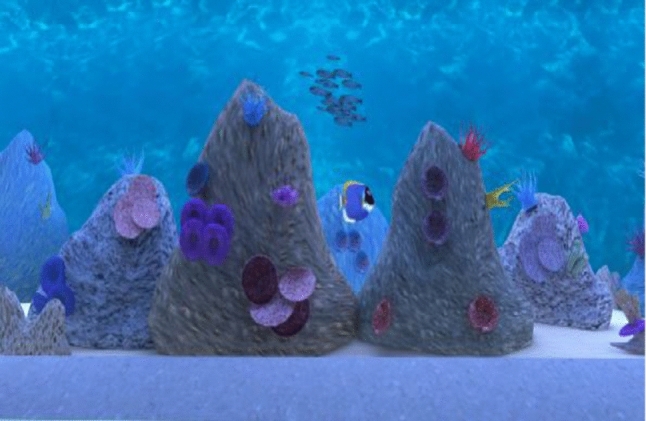
Surgeon fish stimuli

The VR Aquarium system (Climent et al. 2021) consisted of several tasks: (a) a usability task that aimed to allow the participant to familiarize with the task and with the VE. The participant had to find four displays in the VE and position using the VR headset a white dot in the center of the frame of the display by pressing a Flic button; (b) a learning task in which the participant had to press the a Flic button each time they saw or heard the “clown” fish but only if it appeared after they saw/heard the “surgeon” fish, noting that other neutral stimuli/fish are delivered either visually or auditory: “discus,” “bubble,” “angelfish” and “triggerfish.” The task included a total of 160 stimuli (20 for the initial training and 140 for the learning task itself), noting that the data resulted from this task was not used in the data analysis, as its main aim was to train the participant and allow the participant to learn the stimuli; (c) Task 1, dual No_Go task where the user had to press the button each time they saw any other fish than the “clown” or they heard any other fish except the “surgeon.” The training for this task contained 20 stimuli and the task itself had 140 stimuli (words of images of clown, surgeon, discus, bubble, angel and trigger fish) (Figs. 3 and 4) which are distributed in a random order (half of the targets and non-targets are visual and auditory); (d) Task 2, interference dual No_Go task which is similar to the dual No_Go task except that this time the user must press the button whenever they saw any other fish except the “surgeon,” or they heard “clown” fish. Again, the training task comprised 20 stimuli and 140 stimuli for the task itself which were distributed randomly. Only data from dual No_Go task and interference dual No_Go task were used to compute visual and auditory attention scores. To achieve acceptable difficulty and reliability rates, four different versions of the task were developed during the field trials. For the final version, the stimulus interval was set to 500 ms for the visual stimuli and 770 ms for auditory stimuli. Inter-stimuli interval was pseudo-randomized between 1500 and 2000 ms, depending on the item (e.g., visual or auditory stimuli). The total time spent in the VR aquarium was approximately 20–25 min, depending on the user’s accommodation time (see Fig. 2 for Nesplora Aquarium setup).

To increase realism and similarity to real life situations during testing, various contextual distractors were delivered, either visually (e.g., people walking in front of the aquarium, other animals present in the aquarium such as turtles, lights flickering) or audibly (e.g., an invitation to coffee delivered over the PA system, a baby crying, a warning to not use the flash when taking photographs). The total time of the VR task was approximately 20 min, including the warm-up session. Performance was measured by quantifying (a) the reaction (RT) to correct stimuli; (b) number of errors of omission, e.g., not responding to correct targets; (c) errors of commission e.g., responding to incorrect targets. Slower RT reflected slow processing speed, increased number of omission errors indicated inattention and low arousal, and commission errors were linked with increased impulsivity or a deficit in inhibitory control (Climent et al. 2021; Negut et al. 2017; Tinius 2003).

The Nesplora Aquarium used a Samsung Galaxy S7 smartphone, paired with Samsung Gear VR goggles and headphones. The test was monitored by the experimenter, using a laptop computer (ASUS ROG, Intel i7 processor, 8 Gb RAM, GeForce GTX 960 M videocard). Both the laptop computer and the VR headset were connected using a local wireless connection.

### Measures

#### Demographics

Participants’ demographics information was collected using a brief self-report questionnaire. This included age, gender, highest education degree and total years of education, work status, previous VR experience, medical history and whether they experience any severe motion sickness.

#### Self-report system usability

The System Usability Scale (SUS, Brooke 1986) was used as a measure of self-reported system usability and learnability. Usually, the distribution of SUS scores is negatively skewed with a mean around 70 (Bangor et al. 2009, 2008; Brooke 2013), and higher scores represent better performance. For the present study, internal consistency was good with a Cronbach’s *α* of 0.84.

#### Simulator sickness

The level of simulator sickness was measured using the Simulator Sickness Questionnaire (SSQ, Kennedy et al. 1993), one of the most widely used measures of simulator sickness. Higher scores represented increased simulator sickness. To control for potential simulator sickness symptoms at pre-test, we administered a pre-test SSQ and a post-test SSQ to account for post-VR exposure. For the present study, internal consistency was good with α Cronbach of 0.84 (pre-test SSQ) and 0.86 (post-test) SSQ.

#### Presence

To measure the subjective level of presence in VR, we used the Presence Questionnaire version 2 (PQ, Witmer and Singer 1998) with a total of 32 items. PQ was validated on a Romanian population with good internal consistency (Cronbach’s *α* = 0.88) (Opris et al. 2012). Lower scores indicated a diminished sense of presence in the virtual environment.

#### Mental workload

Raw NASA Task Load Index (TLX, Hart and Staveland 1988) was used to measure subjective post-VR exposure workload. We used raw/unweighted scores to compute an overall task load score, as these are as reliable as the weighted scores (Hart 2006). Higher scores indicate an increased estimate of overall workload.

### Procedure

Ethical approval was awarded by the Babes-Bolyai University Research Ethics committee where the data were collected (REF 6667/25.04.2018) and by the University of Bath for data analysis (PREC 18-305). The study was pre-registered on the Open Science Framework (https://osf.io/nrhb5).^2^ Data collection took place between April 2018 and November 2019. After the participants had read the information sheet and before the start of the study, participants gave their written consent. The demographic questionnaire was completed at the beginning of the study. Participants not matching the inclusion criteria did not take part in the study. First, the participants completed the pre-test SSQ. After this, they completed the VR task. During the experience, they were seated in a quiet laboratory space. Participants were instructed to remove the headset and alert the experimenter if they experienced any simulator sickness. At the end of the VR task, the participants filled in paper questionnaires which were administered in a predefined order: post-test SSQ, NASA TLX, PQ and SUS questionnaires. Written instructions were provided at the start of each measure, and additional information concerning completion instructions was provided by the experimenter if required. At the end, they were debriefed. No financial incentives were given for participation.

## Results

Regression analyses were conducted using a bootstrapping procedure with 95% bias-corrected bootstrap Confidence Intervals (CIs) with 10,000 resamples. This method enables more robust CIs and probability values for regression model estimates to overcome issues associated with non-normal distributions (Efron and Tibshirani 1993; Fox 2015; Mallinckrodt et al. 2006). CIs that do not contain zero reflect a significant result. All statistical analyses were performed using IBM SPSS Statistics, version 24 (IBM Corp 2016). The correlation matrix for all the variables is displayed in Table 1.

**Table 1:** Correlation matrix for attention usability, attention performance in VR, demographics, mental workload, presence and simulator sickness

	Mental workload	Presence	Simulator sickness pre-test	Simulator sickness post-test	SUS	RT to correct targets	Omission errors	Commission errors
Age	− .03	− .09	.11	.14	− .37**	.06	.41**	− .01
Previous VR experience	.24*	− .05	− .03	− .08	.13	− .15	− .14	.00
Gender	.12	.16	.02	.15	− .07	.14	.12	− .02
Total years of education	.18	− .11	− .19	− .19	.17	− .02	− .22*	− .05
Mental workload	–	− .09	.06	.33**	− .11	.14	− .10	− .14
Presence	− .09	–	− .11	− .15	.36**	− .07	− .03	.07
Simulator sickness pre-test	.06	− .11	-	.62**	− .26**	.18	.30**	− .19
Simulator sickness post-test	.33**	− .15	.62**	–	− .47**	.18	.37**	− .20
SUS	− .11	.36**	− .26**	− .47**	–	− .24*	− .51**	.19
RT to correct targets	.14	− .07	.18	.18	− .24*	–	− .02	− 42**
Omission errors	− .10	− .03	.30**	.37**	− .51**	− .02	–	− .14
Commission errors	− .14	.07	− .19	− .20	.19	− 42**	− .14	–

Pearson’s r correlation coefficient was computed for numerical variables (age, total years of education, mental workload, presence, simulator sickness, usability, reaction time to correct targets, omission and commission errors; Spearman’s rho correlation coefficient for categorical variables (previous VR experience and gender); **p < .01

### Primary analyses

#### Examined predictors of usability: mental workload, presence and simulator sickness

To investigate our first hypothesis that mental workload, presence and simulator sickness would predict usability of the VR task, while controlling for demographics and VR experience, we conducted a hierarchical regression analysis. Supplementary Table 2 contains means and standard deviations (SDs) for mental workload, presence and simulator sickness. The specific predictors entered into the hierarchical multiple regression model were: age, previous VR experience, gender, total years of education into step 1, and mental workload, presence, simulator sickness at pre-test and at post-test into step 2. Collectively, variables entered into step 1 significantly accounted for 19% of the variance in usability of the VR task, *F* = 4.72, *p* = 0.002, but only age was a significant predictor of usability. When added to step 2, mental workload, presence, simulator sickness at pre-test and at post-test resulted in a significant increase of variance in usability of VR task, *R*^*2*^_change_ = 0.24, *F*_change_ = 7.98, *p* < 0.001 and the full model was significant, *F* = 7.15, *p* < 0.001. As predicted, presence and simulator sickness at post-test were significant predictors of usability when controlling for demographics and VR experience (see Table 2).

**Table 2:** Summary of hierarchical multiple regression results for predicting usability by demographics, mental workload, presence and simulator sickness

Outcome measures	SUS		
Predictors			
	*B*	SE	95% BCa CI [LL, UL]
*Block 1*			
Constant	89.09	9.50	[70.49, 106.68]
Age	− 0.63**	0.19	[− 0.98, − 0.22]
Previous VR experience	0.09	3.30	[− 6.62, 6.91]
Gender	− 3.41	3.13	[− 9.28, 2.28]
Total years of education	0.80	0.60	[− 0.31, 2.00]
R^2^	0.19		
F	4.72**		
*Block 2*			
Constant	60.80	12.83	[36.68, 85.64]
Mental workload	0.00	0.02	[− 0.03, 0.03]
Presence	0.20**	0.06	[0.09, 0.03]
Simulator sickness pre-test	0.23	0.40	[− 0.60, 1.02]
Simulator sickness post-test	− 1.13**	0.44	[− 2.00, − 0.20]
*R*^*2*^	0.42		
Δ*R*^*2*^	0.24		
*F*	7.15***		
Δ*F*	7.98***		

B, unstandardized regression coefficients; SE, standard errors; 95% BCa CI = 95% bias-corrected confidence intervals derived from bootstrapping with 10,000 samples; **p < .01; ***p < .001

#### Examined predictors of performance: mental workload, presence and simulator sickness

Performance in VR was measured with three outcomes: RT to correct responses, omission and commission errors. To test our second hypothesis that mental workload, presence and simulator sickness would predict performance in VR, when controlling for demographics and VR experience, we conducted three hierarchical multiple regression models, one for each outcome. Demographics and previous VR experience were entered at step 1 and mental workload, presence and simulator sickness at step 2. For the first outcome, RT to correct responses, the model was not significant at both step 1 and step 2, *F* = 0.75, *p* = .56, respectively, *R*^2^_change_ = 0.05, *F*_change_ = 1.02, *p* = .40, *F* = 0.89, *p* = .53. For the second outcomes, number of omission errors, collectively, variables entered into step 1 significantly accounted for 24% of the variance, *F* = 6.29, *p* < .001, but only age was a significant predictor. When added to step 2, mental workload, presence and simulator sickness at pre-test and at post-test resulted in a significant increase of variance in usability of VR task, *R*^2^_change_ = 0.10, *F*_change_ = 2.93, *p* = .026 , and the whole model was significant, *F* = 4.91, *p* < .001. The only significant predictor of omissions was simulator sickness at post-test. For the third outcome, the total number of commission errors, at both step 1 and step 2 the model was not significant, *F* = 0.12, *p* = .975, respectively, *R*^2^_change_ = .06, *F*_*change*_ = 1.19, *p* = .32, *F* = 0.66, *p* = .72 (see Table 3).

**Table 3:** Summary of hierarchical multiple regression results for predicting performance in VR by demographics, mental workload, presence and simulator sickness

Outcome measures	Reaction time to correct targets		
Predictors			
	*B*	SE	95% BCa CI [LL, UL]
*Block 1*			
Constant	44.89	6.34	[30.77, 57.55]
Age	0.08	0.15	[− 0.26, 0.35]
Previous VR experience	− 1.37	2.02	[− 5.36, 2.82]
Gender	3.57	2.28	[− 0.42, 7.44]
Total years of education	0.07	0.42	[− 0.75, 0.91]
R^2^	0.04		
F	0.75		
*Block 2*			
Constant	44.68	9.10	[27.71, 60.91]
Mental workload	0.01	0.01	[− 0.01, 0.05]
Presence	− 0.03	0.05	[− 0.13, 0.06]
Simulator sickness pre-test	0.31	0.34	[− 0.30, 0.99]
Simulator sickness post-test	0.02	0.37	[− 0.73, 0.77]
*R*^*2*^	0.07		
Δ*R*^*2*^	0.05		
*F*	0.89		
Δ*F*	1.02		
	Omission errors		
	*B*	SE	95% BCa CI [LL, UL]
*Block 1*			
Constant	46.62	6.15	[34.94, 60.07]
Age	0.46***	0.11	[0.23, 0.65]
Previous VR experience	0.77	2.14	[− 3.27, 5.13]
Gender	0.87	1.82	[− 2.94, 4.94]
Total years of education	− 0.74	0.42	[− 1.57, − 0.00]
*R*^*2*^	0.24		
*F*	6.29**		
*Block 2*			
Constant	43.35	8.14	[26.88, 61.82]
Mental workload	− 0.02	0.01	[− 0.05, 0.01]
Presence	0.01	0.03	[− 0.06, 0.08]
Simulator sickness pre-test	0.08	0.28	[− 0.40, 0.61]
Simulator sickness post-test	0.63*	0.30	[0.06, 1.07]
*R*^*2*^	0.34		
Δ*R*^*2*^	0.10		
*F*	4.91***		
Δ*F*	2.93*		
	Commission errors		
	*B*	SE	95% BCa CI [LL, UL]
*Block 1*			
Constant	53.39	7.11	[40.20, 66.43]
Age	− 0.02	0.11	[− 0.22, 0.23]
Previous VR experience	− 0.79	2.44	[− 5.39, 3.67]
Gender	− 0.97	2.46	[− 5.92, 4.03]
Total years of education	− 0.11	0.37	[− 0.81, 0.27]
*R*^*2*^	0.01		
*F*	0.12		
*Block 2*			
Constant	56.37	13.05	[30.83, 81.11]
Mental workload	− 0.01	0.02	[− 0.04, 0.02]
Presence	0.01	0.05	[− 0.09, 0.11]
Simulator sickness pre-test	− 0.28	0.30	[− 0.89, 0.30]
Simulator sickness post-test	− 0.20	0.33	[− 0.88, 0.50]
*R*^*2*^	0.06		
Δ*R*^*2*^	0.06		
*F*	0.66		
Δ*F*	1.19		

B, unstandardized regression coefficients; SE, standard errors; 95% BCa CI = 95% bias-corrected confidence intervals derived from bootstrapping with 10,000 samples; **p < .01; ***p < .001

#### Relationship between performance and usability

To test our third hypothesis which aimed to identify whether performance in VR is positively associated with usability, we performed a partial correlation while controlling for participants’ demographics and VR experience. The results showed that RT to correct responses and the total number of omission errors were negatively and significantly correlated with self-report usability while controlling for participant demographics (see Table 4).

**Table 4:** Partial correlation between usability and performance in VR by demographics

Outcome measures	SUS	
*Performance in VR*		
Reaction time to correct targets		
	Pearson’s *r*	− .22*
	SE	.10
	95% BCa CI [LL, UL]	[− .38, − .06]
Omission errors		
	Pearson’s *r*	− .38***
	SE	.11
	95% BCa CI [LL, UL]	[− .59, − .13]
Commission errors		
	Pearson’s *r*	.21
	SE	.11
	95% BCa CI [LL, UL]	[− .01, .42]

Demographics (age, gender, previous VR experience and total years of education were controlled for); 95% BCa CI = 95% bias-corrected confidence intervals derived from bootstrapping with 10,000 samples; ***p < .001; **p < .01; *p < .05;

## Discussion

This study used Nesplora Aquarium to examine whether simulator sickness, presence and mental workload predict both usability and attention performance in VR. Simulator sickness and presence significantly predicted usability, but not attention performance. Performance was associated with usability on two out of three outcomes. Findings have several implications for future research investigating the link between usability and attention performance in VR, as well as implications for the design and development of VR applications designed to assess attention, by informing designers of the factors to take into account when designing these tasks in VR.

First, the results revealed that simulator sickness and levels of presence predicted the usability of the VR attention task even when controlling for demographic variables and VR experience. The study results partly support our hypotheses and replicate past work on VR also identifying a positive correlation between presence in VR and usability on other VR tasks than cognitive attention (Brade et al. 2017; Sun et al. 2015; Wienrich et al. 2018). Possibly, because presence is associated with increased enjoyment and psychological involvement leading to exciting feelings in VR, users that experienced increased presence rated the VR system as more usable (Brade et al. 2017; Busch et al. 2014). In the case of simulator sickness, our results are in agreement with previous studies which identified a negative association between simulator sickness and usability (Schultheis et al. 2007; Voinescu et al. 2020). Most likely because simulator sickness distracts the user from the experience, it negatively impacts presence and user experience, including usability (Narciso et al. 2019). These results highlight the importance of reducing simulator sickness symptoms while increasing presence in VR to enhance usability.

In our study, mental workload was not a significant predictor of usability suggesting that the perceived mental workload had no impact on usability ratings. These results contradict other studies that used immersive VR and reported that usability increased with a decrease in mental workload (Kretschmer and Terharen 2019). However, mixed results can be explained by the type of VR environment and tasks used in both studies that may lead to different estimations of mental workload (Kretschmer and Terharen 2019; Rivera-Flor et al. 2019). Kretschmer and Terharen (2019) created an intralogistics immersive gaming experience where participants had to play in immersive VR a typical task of packaging (e.g., selecting a box, placing items in a box) with a mean workload of 30.06 vs. 41.97 in our study. The mental workload in the present study, as measured with Raw NASA TLX, was 41.97 (SD = 14.07). This is slightly lower than the average NASA TLX scores (*M* = 45.29) reported in a review of 33 studies (Grier 2015). Also, in terms of usability ratings our system (*M* = 80.17, SD = 15.19) was excellent according to cutoff scores provided in the literature (Bangor et al. 2009, 2008). Our ratings of usability were slightly higher than other self-report usability ratings provided in the literature for non-immersive or immersive VR applications (Corno et al. 2014; Fang and Lin 2019; Napa et al. 2019; Pedroli et al. 2013).

Importantly, age was also a significant predictor of usability. The younger the participants were, the more likely they were to rate the VR system as more usable and committed less omission errors. Our results are in accordance with other studies which showed that younger participants compared to older participants perform better on VR tasks (e.g., website navigation, navigation in desktop virtual environments or Nintendo Wii exergaming system) (Meldrum et al. 2012; Sayers 2004; Wagner et al. 2014). Computer knowledge and experience can negatively influence task performance (Jacko et al. 2004). Older adults are less likely to adopt new technology than younger adults (e.g., technology in general, computers and the Internet), noting that cognitive abilities mediate this relationship (Czaja et al. 2006). Healthy cognitive aging is associated with a decrease in cognitive functioning (e.g., spatial ability or attention) that might impact their performance and usability ratings (Wagner et al. 2014). This is the reason why it is widely recognized in the HMI field that interface designs should accommodate individuals with a diverse range of needs and abilities, including older adults and clinical populations (Fisk et al. 2009).

For attention performance on the VR task, contrary to expectation the only significant predictor of omission errors was simulator sickness. Omission errors are an indicator of inattention and low arousal (Climent et al. 2021). This suggests that experiencing increased symptoms of simulator sickness may contribute to increased inattention and low arousal during VR testing. We mention that the mean of simulator sickness reported by our participants was 5.78 (SD = 5.13), which means that our participants reported, on average, minimal symptoms (Stanney et al. 1997). Despite a low degree of simulator sickness, in the current study we highlight that it was the only factor that predicted both usability and task performance. Our study tested the effects of simulator sickness on attention performance in real time during the VR task rather than at the end of it which has the advantage of capturing in real time any negative effects and eliminates time-related confounds (e.g., fatigue post-VR exposure). In relation to RT, our study did not find a negative relationship between simulator sickness and reaction time, in contrast to previous studies which used RT measures obtained after VR exposure (Mittelstaedt et al. 2019; Nalivaiko et al. 2015; Nesbitt et al. 2017), suggesting that during the VR exposure, no significant changes in RT occur. However, it is important to note the low levels of simulator sickness within this study that can also explain the non-significant results.

Other human factors such as presence in VR and mental workload were not associated with attention performance. Although previous research reported mixed findings concerning the relationship between presence and task performance, our study used an attentional VR task to examine this relationship (Cooper et al. 2018; Makransky et al. 2019; Price et al. 2011; Rose and Chen 2018; Stevens and Kincaid 2015). What these contradictory results suggest is that presence may or may not predict performance in VR depending on the environment and task used, given that features related to VR technology such as interactivity, fidelity and level of engagement can increase presence (Piccione et al. 2019) and vary in the aforementioned studies. In our study, participants experienced the virtual environment (the aquarium) passively as the task did not require active navigation and movement in it, while research showed that active VR experiences can lead to more enhanced presence than passive VR experiences (Piccione et al. 2019; Sheridan 1992; Witmer and Singer 1998). However, a significant association between lower levels of presence than in our study (*M* = 119, SD = 18.26 vs *M* = 155.09, SD = 23.55) and performance was reported by (Stevens and Kincaid 2015). In relation to mental workload, we expected a significant relationship with attention performance because experiencing either increased workload or reduced workload affects task performance, as highlighted in previous non-VR studies (Longo 2018). To the contrary, our results suggest that self-reported levels of mental workload have no major impact on performance. In our study, the association between usability and performance revealed slightly linear associations for RT and commission errors and a slightly U-shaped distribution for omission errors (see Supplementary materials). This suggests that both low and high levels of mental workload would negatively impact performance, while the optimum level of performance would be at moderate level of mental workload.

Our findings partially supported our hypothesis that attention performance in VR would be associated with usability when controlling for participants’ demographics. In our study, we had three outcome variables that measured performance on the VR task: RT to correct responses, omission and commission errors. Slower RT reflects slow processing speed, increased number of omission errors indicates inattention and low arousal, and commission errors are linked with increased impulsivity or a deficit in inhibitory control (Climent et al. 2021; Negut et al. 2017; Tinius 2003). Our results indicated that RT to correct responses and omissions were negatively associated with usability after controlling for participants demographics. These results suggest that for this attention task participants that considered the Nesplora Aquarium system as really usable also had better attention performance on the VR task, mainly better reaction time and increased arousal and lower inattention, i.e., by increased inattention and low arousal during the VR task and faster RT times to correct targets. On the other hand, we did not identify a significant association between commission errors and usability, meaning that the link between inhibitory control in VR and usability was not proven for the current VR attention task. Overall, for the relationship between usability and attention performance, some of our results replicated findings from the literature that range from strong and positive correlations between usability and performance (Nielsen and Levy 1994) to weak correlations (Hornbæk and Law 2007) on a wide range of systems other than VR. Similar to the mixed results reported in the current study, a recent study that used an immersive VR supermarket delivered via an HMD where participants had to perform various shopping tasks showed a mixed pattern of results for the relationship between usability and performance (Mondellini et al. 2018). For example, authors reported no significant association between usability and errors committed, while time needed to complete the task predicted usability which was replicated in the current study. Heterogeneity in these results might be explained by various tasks, performance metrics (e.g., time-based or errors-based), HCI systems assessed (e.g., web platforms, interface types such as touchscreen or desktop, mouse and keyboard), and complexity of usability measures (Hornbæk and Law 2007). We suggest that the VR system we used in the present study to assess attention was more complex than the systems used in previous studies (e.g., websites or early computer interfaces), and task complexity (e.g., skill based tasks linked with low complexity, rule-based tasks with moderate complexity, knowledge-based tasks as highest in complexity) has been shown to reduce the magnitude of correlations between objective and subjective usability (see Hornbæk and Law 2007 for a review).

Mental health care is a key area in which VR shows increased potential (e.g., treating phobias or assessment and rehabilitation of cognitive functioning) (Freeman et al. 2017; Rizzo et al. 2019). The VR task used in the current study was developed to measure attention performance with the aim of detecting possible attention impairments among healthy and/or clinical populations (e.g., depression, anxiety) (Voinescu et al. 2021). Because increased usability might improve acceptance rates among users and increase adherence to VR-based therapies (Laver et al. 2012; Moran et al. 2015), the results of the current study can inform the development of VR-based interventions in clinical psychology and suggest that for clinical VR-based applications more emphasis should be placed on creating environments and tasks that can increase a sense of presence and reduce simulator sickness to increase usability. The focus on these variables can be especially important for adherence to a treatment and limit dropouts but that the influence on performance at least in terms of attention may be minimal.

## Limitations

One of the limitations of our study is that our results may not generalize to other tasks as we specifically assessed attention using a passive virtual experience designed to be used eventually with clinical populations. Future studies could investigate the effect of mental workload, presence and simulator sickness on other cognitive tasks in VR such as learning, memory or spatial navigation using active and passive exploration of the virtual environment.

In our study, we identified a negative effect of age on usability and attention performance in a sample of adults younger than 65. Future studies may investigate this effect using older participants, as older populations might be one key category of users who could benefit from various VR applications. For example, VR applications are being tested with older adults to see whether they can decrease risk of falls, pain or improve cognition and well-being (Dermody et al. 2020; Karssemeijer et al. 2019a, b; Lee et al. 2019; Skurla et al. 2022). In the current study, due to ethical considerations we excluded participants with self-reported severe motion sickness in an attempt to avoid severe symptoms during the VR task since motion sickness is a predictor of simulator sickness (Golding et al. 2021; Kennedy and Fowlkes 1992). A potential drawback of this could be the selection of participants with low or moderate scores that are not representative of the whole population which could limit the generalizability of our results and representativeness of the data. However, participants with high levels of motion sickness and plausibly simulator sickness would not be eligible for VR interventions and experiences as the negative effects would overcome the benefits.  

## Conclusions

The results of the current study can enhance the current understanding concerning predictors of both usability and attention performance and the relationship between usability and attention performance in immersive VR. This study has shown that presence and simulator sickness are features of VR that impact usability. Experiencing simulator sickness was also related to worse attention task performance but there is less evidence for this as only one performance outcome was negatively impacted by simulator sickness. With regard to the relationship between self-report usability and attention task performance, our study finds that the two constructs most likely correlate with each other, suggesting that for attention VR applications people are more likely to rate the applications where they perform well as more usable. Although our study did not test whether over time the link between usability and attention performance would strengthen, our findings suggest that achieving high level of usability to facilitate product uptake may result in better performance or vice versa.

## Supplementary Information

Below is the link to the electronic supplementary material.

Supplementary file1 (DOCX 92 KB)

## Data Availability

The data that support the findings of this study are available from the corresponding author, Alexandra Voinescu, upon reasonable request.

## Footnotes

1 In our sample, we did not include older adults as defined by WHO (age higher than 65 years (WHO 2015) to avoid any potential confounds related to age, e.g., aging can be associated with cognitive decline (Deary et al. 2009).

2 The current study was part of a larger program of work that aimed to establish the validity and clinical effectiveness of the Nesplora Aquarium in discriminating on attention measures between healthy controls and people with cognitive impairment caused by depression and anxiety disorders.
