# Supplementary material for: Presence and simulator sickness predict the usability of a virtual reality attention task
Source: Virtual Real. 2023 Mar 24:1–17. Online ahead of print. doi: 10.1007/s10055-023-00782-3 (PMC10038382; doi:10.1007/s10055-023-00782-3)

## Supplementary materials

| Supplementary Table 1. *Demographic characteristics* | |
| --- | --- |
|  | Total |
| Age (*M*) | 31.81 (*SD*=9.78) |
| Gender  Male  Female | *N*  34 (39.1%)  53 (60.9%) |
| Education | *N* |
| Primary school | 1 (1.1%) |
| Secondary school and vocational training | 26 (29.9%) |
| First degree (e.g. bachelor studies) | 27 (31%) |
| Higher degree (e.g. Master, PhD) | 33 (37.9%) |
| Work status  Employed  Not employed | *N*  54 (62.1%)  33 (37.9%) |
| Previous VR use/experience  Yes  No | *N*  35 (40.2%)  52 (59.8%) |

| Supplementary Table 2. *Means and SDs for* *system usability,* *mental workload, presence and simulator sickness* | | |
| --- | --- | --- |
| Outcomes | *M* | *SDs* |
|  |  |  |
| System usability | 80.17 | 15.19 |
| Mental workload (sum of subscales) | 251.84 | 84.39 |
| Mental workload (average of subscales) | 41.97 | 14.07 |
| Presence | 155.09 | 23.55 |
| Simulator sickness at pretest | 5.32 | 4.93 |
| Simulator sickness at posttest | 5.78 | 5.13 |
| *Note.* System usability was measured with SUS; Mental workload was measured with Raw Nasa TLX; Presence was measured using PQ; Simulator sickness was measured using SSQ | | |

# Results

## Scatter plots for self-report usability and task performance

A visual inspection of the plots for the association between usability and task performance revealed slightly linear associations for RT and commissions and a slightly U-shaped distribution for omission errors, which could explain the non-significant association identified through the regression analysis (see Supplementary materials). More specifically, the U-shaped distribution in case of mental workload and omission errors would indicate that both low and high levels of mental workload would impact negatively performance while the optimum level of performance would be at moderate level of metal workload.


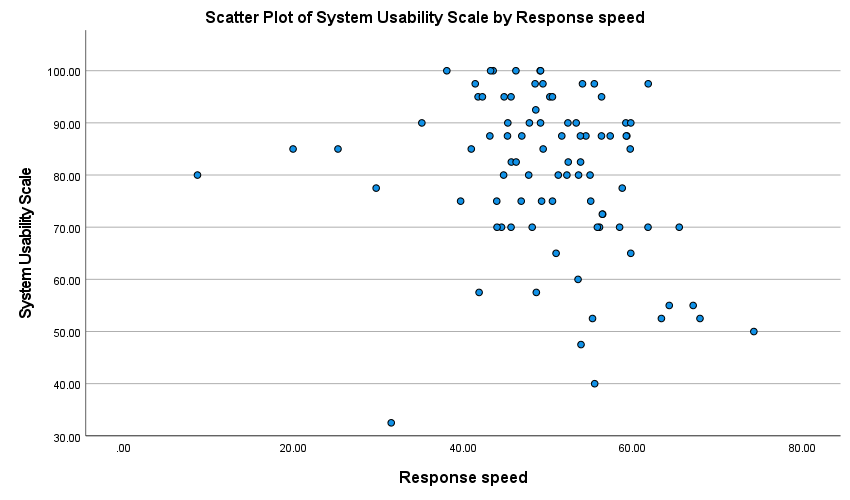


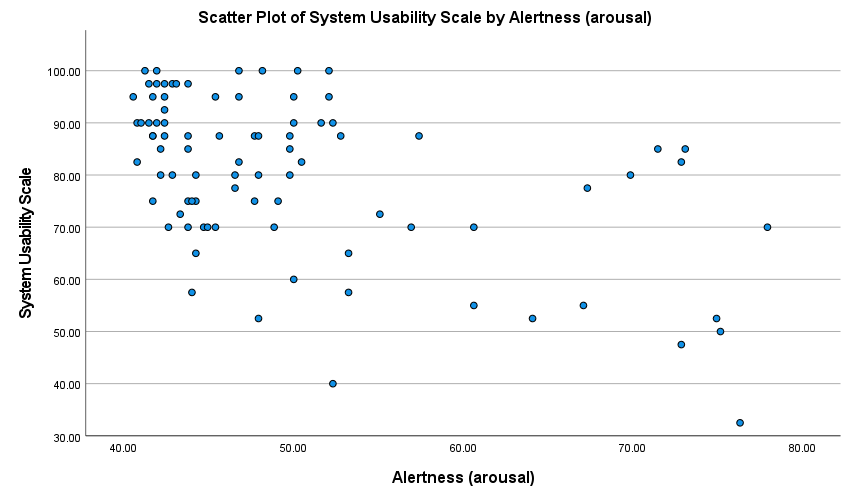


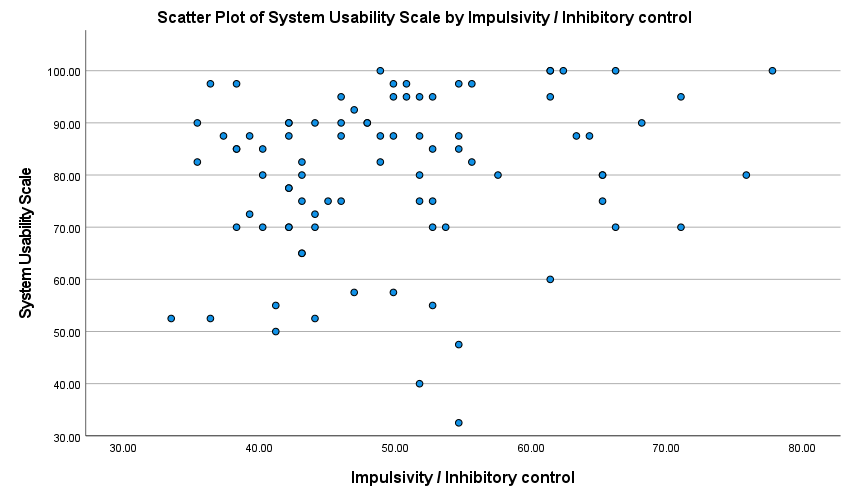

Supplement: Supplementary file 1 — Supplementary file1 (DOCX 92 KB) [file 10055_2023_782_MOESM1_ESM.docx]
